# Pulchrin A, a New Natural Coumarin Derivative of *Enicosanthellum pulchrum*, Induces Apoptosis in Ovarian Cancer Cells via Intrinsic Pathway

**DOI:** 10.1371/journal.pone.0154023

**Published:** 2016-05-02

**Authors:** Noraziah Nordin, Mehran Fadaeinasab, Syam Mohan, Najihah Mohd Hashim, Rozana Othman, Hamed Karimian, Venus Iman, Noorlela Ramli, Hapipah Mohd Ali, Nazia Abdul Majid

**Affiliations:** 1 Department of Pharmacy, Faculty of Medicine, University of Malaya, Kuala Lumpur, Malaysia; 2 Department of Chemistry, Faculty of Science, University of Malaya, Kuala Lumpur, Malaysia; 3 Medical Research Center, Jazan University, Jazan, Kingdom of Saudi Arabia; 4 Institute of Biological Sciences, Faculty of Science, University of Malaya, Kuala Lumpur, Malaysia; Institute of Biochemistry and Biotechnology, TAIWAN

## Abstract

Drug resistance presents a challenge in chemotherapy and has attracted research interest worldwide and particular attention has been given to natural compounds to overcome this difficulty. Pulchrin A, a new compound isolated from natural products has demonstrated novel potential for development as a drug. The identification of pulchrin A was conducted using several spectroscopic techniques such as nuclear magnetic resonance, liquid chromatography mass spectrometer, infrared and ultraviolet spectrometry. The cytotoxicity effects on CAOV-3 cells indicates that pulchrin A is more active than cisplatin, which has an IC_50_ of 22.3 μM. Significant changes in cell morphology were present, such as cell membrane blebbing and formation of apoptotic bodies. The involvement of phosphatidylserine (PS) in apoptosis was confirmed by Annexin V-FITC after a 24 h treatment. Apoptosis was activated through the intrinsic pathway by activation of procaspases 3 and 9 as well as cleaved caspases 3 and 9 and ended at the executioner pathway, with the occurrence of DNA laddering. Apoptosis was further confirmed via gene and protein expression levels, in which Bcl-2 protein was down-regulated and Bax protein was up-regulated. Furthermore, the CAOV-3 cell cycle was disrupted at the G0/G1 phase, leading to apoptosis. Molecular modeling of Bcl-2 proteins demonstrated a high- binding affinity, which inhibited the function of Bcl-2 proteins and led to cell death. Results of the current study can shed light on the development of new therapeutic agents, particularly, human ovarian cancer treatments.

## Introduction

Cancer is a major disease affecting the human population worldwide [1]. Approximately, half of all men and more than one-third of all women are diagnosed with cancer over the course of their lifetime. Meanwhile, one-quarter of adults die because of cancer [2]. Data compiled by the International Agency for Research in Cancer (IARC) on cancer registration and mortality show that nearly 12.6 million new cancer cases were reported in 2008 alone worldwide [2]. According to the National Cancer Registry of Malaysia [3], a total of 8,123 (44.6%) males and 10,096 (55.4%) females residents were diagnosed with cancer in Peninsular Malaysia in 2007.

Meanwhile, a total of 239,000 new cases worldwide were recorded for ovarian cancer [4]. Ovarian cancer is the most fatal gynaecological cancer mainly because of the lack of symptoms specificity and biomarkers available for detection during the early stages of the disease. In the majority of ovarian cancer cases, late-stage diagnosis was commonly detected among patients who were unable to effectively respond to the treatment. Generally, these patients have a 5-year survival rate, but this rate has been reduced to 20–30% [5–7]. Treatment of patients with ovarian cancer is based on the standard protocol whereby surgery is the initial treatment followed by chemotherapy. Three different drugs commonly used to treat ovarian cancer are doxorubicin, carboplatin and taxane. However, these drugs are often less effective whereby patients may exhibit resistance to the administered drug [8]. These disadvantages have prompted researchers to explore potentially effective alternative compounds as treatment for ovarian cancer.

Coumarin and its derivatives belong to the lactone family comprising the benzopyrone skeletal framework, which can be found widely in nature [9]. Coumarin derivatives have been found to exhibit considerable therapeutic and various biological activities [10, 11] that are useful in photochemotherapy, antitumor therapy and anti-HIV therapy [12, 13]. They can be used as central nervous system (CNS) stimulants [14], antibacterials [15, 16], antifungals [17, 18], anti-inflammatories [19], anti-coagulants [20], tuberculostatics [21] and dyes [22]. Some of coumarin derivatives have also been reported as fixatives and flavoring agents. However, the United States Food and Drug Administration (FDA) has regulated the use of coumarin as food additives [23–25]. Potent antibiotics derived from coumarin, such as novobiocin, coumaromycin and chartesium are commercially available [26]. In the present study, a new coumarin derivative was isolated for the first time from natural product, *Enicosanthellum pulchrum*. A long alkyl group was connected to heterocyclic coumarin ring to investigate its potential as promising anticancer agent against the human ovarian cancer cell line CAOV-3 at low concentrations within 24 h.

## Materials and Methods

### Experimental

Silica gel H was purchased from Sigma-Aldrich, whereas silica gel 60 (230˗400 mesh), dimetylsulfoxide (DMSO), methanol (MeOH), ethanol (EtOH), *n*-hexane and ethyl acetate (EtOAc) were purchased from Merck Co. (Germany). 3-(4,5-Dimethylthiazol-2yl)-2, 5-diphenyl tetrazolium bromide (MTT reagent) was provided by Invitrogen (Carlsbad, USA). RPMI-1640 medium (pH 7.4), penicillin/streptomycin and fetal bovine serum (FBS) were purchased from Nacalai Tesque (Japan) and Trypsin-EDTA 10X was supplied by Biowest (USA). The ultraviolet (UV) spectra and the Infrared (IR) spectra were recorded on a UV spectrophotometer (UV-1601 Shimadzu, Japan) and the FT-IR spectrometer Spectrum RXI (Perkin Elmer, USA), respectively. The nuclear magnetic resonance (NMR) spectra were recorded on a 600 MHz Bruker (Switzerland) spectrometer. Optical rotation was performed in Jusco (Tokyo, Japan). Mass spectra were recorded for electron spray ionization (ESI) mass spectrometry on IT-TOF from Shimadzu, Japan. High-performance liquid chromatography (HPLC) analysis was also conducted.

### Preparation of plant extraction

Roots of *E*. *pulchrum* were collected in September 2011 at the mountain forest of Cameron Highlands (Pahang, Malaysia). The Director of the Forestry Department of Pahang, Malaysia was given the permission to enter and collect the samples [27]. The plant was identified by the late Prof. Dr. Kamarudin Mat Salleh from Universiti Kebangsaan Malaysia (UKM). The specimen (SM769) was placed at the Botany Department Herbarium, Faculty of Science and Technology, UKM (Bangi, Malaysia). The roots were air-dried and ground to 40–60 mesh particle size. The extracts were obtained by maceration in *n*-hexane solvent. A rotary evaporator (Buchi, Switzerland) was used to remove the solvents from the samples.

### Extraction and isolation of pulchrin A

For purification of pulchrin A, the hexane extract was separated by column chromatography (CC) using silica gel type 60 (230–400 mesh). The solvent system was used to separate the compounds in the gradient column from less polar to most polar (hexane-EtOAc-MeOH). A total of 157 fractions were collected using a 20 mL vial. A total of 12 fractions were obtained based on the retention factor of each compound by thin-layer chromatography (TLC) analysis. Fraction 3 (A9–A12) was further purified using prep-TLC. A new compound was successfully separated using the *n*-hexane-EtOAc (8:2) solvent system. The compound referred to as pulchrin A was elucidated by NMR, whereas the purity of pulchrin A was determined by HPLC using 10% to 100% acetonitrile (v/v) gradient elution over 15 min at a flow rate of 0.5 mL/min.

### Structural Elucidation Analyses

#### Nuclear Magnetic Resonance Spectroscopy (NMR)

In brief, pulchrin A was analyzed by NMR to examine the presence of proton and carbon atoms through 1D (^1^H and ^13^C) and 2D (COSY-45, HSQC and HMBC) experiments. The compound was prepared by dissolving with deuterated chloroform (CDCl_3_) in the NMR tube. Prior to analysis, the NMR tube was placed in the NMR spectrometer for further processing.

#### Mass Spectroscopy (MS)

Mass spectroscopy is mainly to determine the molecular weight of pulchrin A. The analysis was performed utilizing the LCMS-IT-TOF instrument. Pulchrin A was dissolved in MeOH prior to injecting into the instrument. The mass spectrum was recorded on the positive and negative mode.

#### Fourier Transform-Infrared Spectroscopy (FT-IR)

The Fourier Transform-Infrared spectroscopy (FT-IR) was conducted to detect the presence of functional group in pulchrin A. The compound was dissolved in CHCl_3_ and dropped onto the sodium chloride (NaCl) pellet. Prior to analysis, the pellet was placed in the FT-IR instrument. The result was recorded at the absorption of 500–4000 cm^-1^.

#### Ultraviolet Spectroscopy (UV) and Optical Rotation

Pulchrin A was dissolved in MeOH prior to being transferred into a cuvet. The wavelenghts used to detect the presence of atoms, ranged from 190 nm to 800 nm. The absorption was then recorded using an UV spectrophotometer. Results were expressed in the form of an absorption spectrum. Meanwhile, a total of 0.05 M of pulchrin A was dissolved in CHCl_3_ and transferred into a Jasco P-1020 polarimeter. The temperature of measuring the optical rotation was 24°C.

### Cytotoxicity assay

Two human ovarian cancer cells (CAOV-3 and SKOV-3) were originally purchased from American Type Culture Collection (ATCC, Manassas, USA). Immortalized human ovarian epithelial cells (T1074) were obtained from Applied Biological Materials (ABM^®^ Crestwood Place Richmond, Canada). These cell lines were cultured in our institution laboratory. All cells were subcultured and grown in 25 mL flask containing RPMI-1640 medium (CAOV-3 and SKOV-3) and Prigrow 1 medium (T1074) [28], complemented with 10% FBS and 1% penicillin/streptomycin. Confluent cells were then washed with PBS before being harvested with Trypsin-EDTA 10X solution. The harvested cells were centrifugated at 1800 rpm for 5 min and diluted to 1 × 10^6^ cells/mL cells. Assay was performed in a 96-well plate containing 1 × 10^4^ cells/well. Prior to treatment, a total of 1 × 10^4^ μg/mL of pulchrin A was prepared by adding 1.0 mg of compound in 100 μL of DMSO. The CAOV-3 cells were treated with pulchrin A at the concentration of 50 μg/mL up to 0.78 μg/mL by 2-fold serial dilution. The cells was then incubated at 24, 48 and 72 h. Prior to measurement, MTT solution (20 *μ*L) was added in each well and incubated for 3 h. The plate was recorded at an absorbance of 570 nm by using a microplate reader. The result was set as the IC_50_ value, whereby cisplatin (IC_50_: 30.6 μM) was used as positive control in the study.

### Acridine orange/propidium iodide (AO/PI) double staining assay

AO/PI double staining assay was conducted in accordance with the standard procedure used for fluorescence microscopy (Lieca with the Q-Floro software). The assay was performed in a 25 mL culture flask (Nunc), whereby the CAOV-3 and T1074 cells were exposed with pulchrin A (22 μM) at 24, 48 and 72 h. Prior to staining, the harvested cells were washed with PBS. A total of 10 *μ*L of AO and PI (10 *μ*g/mL) were added to the cell pellet. The cells were observed within 30 min under a UV-fluorescent microscope (Olympus BX51) to evaluate the morphological changes before fluorescence fading.

### Annexin-V-FITC assay

The effect of pulchrin A during the initial stage of apoptosis was assayed using the fluorescein isothiocyanate (FITC) Annexin V Apoptosis Detection Kit I (BD Pharmingen^™^). The CAOV-3 cells were grown into a 6-well plate and treated with pulchrin A (22 μM) for 24, 48 and 72 h. The cells (5 × 10^4^ cells/mL) were then collected by centrifuging at 1600 rpm for 5 min. A total of 100 μL of each sample was transferred into a tube containing 5 μL of FITC Annexin V and 10 μL of PI. The suspension was mixed gently and added with 100 μL of 1 × assay buffer. Detection was performed using a flowcytometer (BD FACSCanto^™^II, San Jose, CA, USA).

### Colorimetric caspases analysis

Cysteinyl aspartic acid-protease (Caspases) -3, -8 and -9 assays were conducted using a commercial kit (caspases 3, 8 and 9 colorimetric assay: R&D Systems, Inc. USA). The cells were seeded in a 75 mL flask and treated with the IC_50_ concentration of pulchrin A for 24, 48 and 72 h. The treated cells were collected by centrifugation at 1800 rpm for 5 min. The lysis buffer was then added to the cell pellet and incubated on ice for 10 min. The lysate was further centrifuged at 9450 rpm for 1 min to collect the supernatant. The assay was conducted in a flat bottom 96-well microplate. Approximately 5 μL of caspase 3, 8 or 9 colorimetric substrate (LEHD-pNA) was added to each reaction containing a cell lysate and a 2 X reaction buffer of caspase 3, 8 or 9. The reaction was incubated for 1 h at 37°C and then read on a microplate reader (Infinite M200PRO, Tecan, Männedorf, Switzerland) at wavelength of 405 nm.

### Multiple cytotoxicity assays

Multiple cytotoxicity assays were conducted using the Cellomics^®^ Multiparameter Cytotoxicity 3 Kit (Thermo Scientific, PA, USA). A total of 5 × 10^3^ cells were seeded in each well of 96-well microplate. The cells were then treated with pulchrin A at three concentrations (11, 22 and 33 μM) for 24 h and then incubated overnight at 37°C. After incubation, several solutions were continuously added in each well containing 50 μL of live cell staining solution, 100 μL of fixation buffer, 100 μL of 1 X permeabilization buffer and 100 μL of 1 X blocking buffer, which were incubated for 20 min, 10 min, 30 min and 15 min, respectively. Two antibody solutions (cytochrome *c* primary antibody and DyLight^™^ 649 Conjugated Goat Anti-Mouse IgG secondary antibodies) were added at the final stage of assay preparation. The plate was then read and evaluated on the ArrayScan, high content screening (HCS) Reader from Thermo Fisher Scientific (Pittsburgh, PA, USA).

### DNA fragmentation assay

This experiment was conducted using a Suicide-Track^™^DNA Ladder isolation Kit (Calbiochem, KgaA, Darmstadt, Germany). The COAV-3 cells were seeded into 75 mL culture flasks and then treated with pulchrin A (22 μM) for 24, 48 and 72 h. Cell pellets were obtained by centrifugation at 1800 rpm for 5 min. The cell pellets were gently resuspended in three solutions successively: 55 μL of solution #1, 20 μL of solution #2 and 25 μL of solution #3 (kit components). Prior to incubation at 50°C, 500 μL of the resuspension buffer was added into the mixture. Electrophoresis was performed by preparing the agarose gel (1.5%) in 1 X TAE buffer with a staining reagent supplied in the kit. The gel was run at 50 volts until the dye reached 1–2 cm from the end of the gel. The gel was visualized under a UV light transilluminator and then photographed.

### Real-time PCR

Total RNA was extracted from CAOV-3 cells by using RNeasy Mini Kit (Qiagen, Germany). The final concentration of total RNA and its purity were measured using a Nanodrop 2000 spectrophotometer (Thermo Scientific). Conversion from RNA to cDNA was performed using the Two-Step qRT-PCR Kit (Applied Biosystems, USA). A total of 1 μg/mL of cDNA (1 μL) was used in the genes expression assay with seven TaqMan specific primers and probe (β-actin, Bax, Bcl-2, survivin, as well as caspases 3, 8 and 9) genes were added in the assay (Applied Biosystem, USA). The gene expression levels were then evaluated using the StepOne Plus Real-time PCR System (Applied Biosystems, USA); each cycle consisted of 2 min of reverse transcriptase at 50°C, 20 sec of polymerase activation at 95°C, 1 sec of denaturation at 95°C and 20 sec of annealing at 60°C. The process was completed after 40 cycles of reading. Data were then calculated based on the comparative Ct (2-ΔΔCt) standard method described in the study by Wong and Medrano (2005) [29].

### Western blot assay

The CAOV-3 cells were seeded in a 75 mL culture flask and treated with pulchrin A (22 μM) at 24, 48 and 72 h. The harvested cells were centrifuged at 13,000 rpm for 10 s and 400 μL of PRO-PREP^™^ solution was added to resuspend the cells. The cells were induced for lysis by incubation at -20°C for 20 min. Prior to separation by 10% SDS-PAGE, 100 μg of protein was aliquoted and mixed with loading dye. The gel was allowed to run for 90 min before it was transferred to a polyvinylidenedifluoride (PVDF) membrane (Bio-Rad). The PVDF membrane was blocked for 3 h with 5% BSA. The primary antibody for *β*-actin (1: 1000), Bax (1: 1000), Bcl-2 (1:1000), survivin (1:1000), caspases 3 and 9 (1:1000) and cleaved caspases 3 and 9 (1:1000) were conjugated with a secondary antibody (Goat pAb to Rb IgG). The process continued for 1 h incubation at room temperature. The bound antibody was detected using a colorimetric detection kit and exposed for several minutes to allow the appearance of the bands. The PVDF membrane was viewed and photographed using a UV light transilluminator.

### Protein profile array

CAOV-3 cells treated with Pulchrin A (22 μM) were evaluated for apoptotic protein markers by using the Proteome Profiler Array (RayBio^®^ Human Apoptosis Antibody Array Kit, Raybiotech, USA), in accordance with the manufacturer’s protocol. Extracted protein (200 μg/mL) from CAOV-3 cells was added into each well containing membrane and was left overnight at 4°C for incubation. The washing procedure using wash buffers I and II was executed for each incubation. Prior to detection, the membrane was added with a biotinylated antibody cocktail and subsequently with HRP-streptavidin for incubation overnight. A total of 500 μL of the detection buffer was pipetted onto the membrane. The sandwiched membranes were transferred and exposed to the chemiluminescence imaging system and then photographed (Biospectrum AC Chemi HR 410, UVP, Cambridge, UK).

### Cell cycle assay

The treated cells with pulchrin A were collected and then washed twice with PBS at 1800 rpm centrifugation for 5 min. The cells were fixed with a fixation solution by adding 700 μL of 90% cold EtOH and kept overnight at 4°C to restore cells integrity. EtOH was then discarded and rinsed with 600 μL of PBS by centrifugation at 1800 rpm for 5 min. A total of 25 μL of RNaseA (10 mg/mL) and 50 μL of propidium iodide (PI) (1 mg/mL) were mixed to the fixed cells and incubated for 1 h at 37°C. Analysis of the DNA content for cell cycle arrest was performed by flowcytometry (BFACSCanto^™^II).

### Protein-binding interaction

This study was performed using the anti-apoptotic protein Bcl-2 in conjuction with the standard Bcl-2 inhibitor ABT 737 [30]. The Bcl-2 three-dimensional crystal structure was obtained from the RCSB Protein Data Bank [31, 32] with 4IEH PDB id’s [30]. The automated docking software (AutoDock 4.2) program was used to calculate the docking of small molecules, based on the Lamarckian genetic algorithm [33]. Water molecules were removed for the preparation of protein molecules. Polar hydrogen atoms were added into the structure, whereas nonpolar hydrogen atoms were merged. In addition, Gasteiger charges and solvation parameters were assigned by default. By using the AutoGrid 4.2 software, the grid parameter file was set using values for x, y, and z axes; the grid spacing was to 0.375 Å, the default value. The interaction binding energy was also calculated using the AutoDock 4.2 program.

### Statistical analysis

The IC_50_ values were determined using GraphPad Prism ver. 4.0 (Graphpad Software Inc, USA). Each test was conducted in three replicates and the values were reported as mean ± standard deviation (SD). By using SPSS ver. 17.0 (IBM Corporation, USA), one-way ANOVA with Dunnett’s test was used to analyze the data for statistical significance. Meanwhile, quantitative analyses of proteins were perfomed using ImageJ software.

## Results

### Structure Identification of Pulchrin A

**Fig 1:**
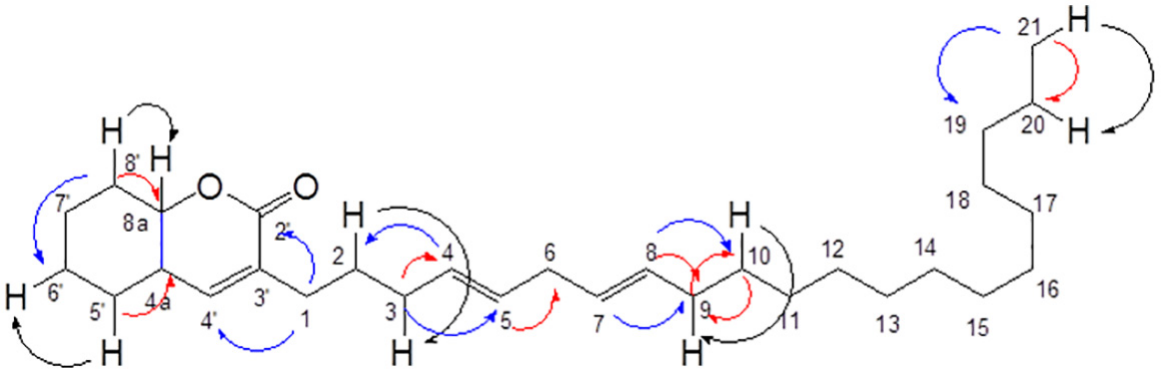
Structure of pulchrin A with selected HMBC and COSY correlation. The HMBC correlation is highlighted with red and blue arrows indicated as *J*_2_ and *J*_3_, respectively, while COSY correlations in black arrow color.

A new natural coumarin derivative, pulchrin A (Fig 1) was elucidated by using the complete NMR spectra (Table 1), as well as UV, IR and ESI mass spectrometry (S1–S7 Figs).

**Table 1:** Spectroscopic data of 1D and 2D-NMR in chloroform-*d* for pulchrin A (δ in ppm, *J* in Hz).

H/C	^δ^H in ppm (*J* in Hz)^a^	^δ^C in ppm^b^	HMBC
1	2.25 (2H, *m*, CH_2_)	25.2	2´, 4´
2	1.51 (2H, *m*, CH_2_)	26.6	-
3	2.21 (2H, *m*, CH_2_)	24.3	4, 5
4	5.40 (1H, *m*, CH)	131.0	2
5	5.40 (1H, *m*, CH)	131.0	-
6	2.91 (2H, *m*, CH_2_)	57.3	-
7	5.33 (1H, *m*, CH)	129.8	9
8	5.35 (1H, *m*, CH)	128.3	9, 10
9	2.02 (2H, *m*, CH_2_)	27.4	-
10	1.55 (2H, *m*, CH_2_)	28.0	9
11	1.28 (2H, *m*, CH_2_)	29.5	-
12	1.28 (2H, *m*, CH_2_)	29.5	-
13	1.28 (2H, *m*, CH_2_)	29.5	-
14	1.28 (2H, *m*, CH_2_)	29.5	-
15	1.28 (2H, *m*, CH_2_)	29.6	-
16	1.28 (2H, *m*, CH_2_)	29.6	-
17	1.28 (2H, *m*, CH_2_)	29.6	-
18	1.25 (2H, *m*, CH_2_)	22.7	-
19	1.30 (2H, *m*, CH_2_)	32.0	-
20	1.32 (2H, *m*, CH_2_)	22.4	-
21	0.89 (3H, *m*, CH_3_)	14.1	19, 20
2´-O-C = O	-	173.9	-
3´	-	134.4	-
4´	6.98 (1H, *m*, CH)	148.8	2´, 3´, 8a
4a	2.93 (1H, *m*, CH)	56.8	5´
5'	1.59 (2H, *m*, CH_2_)	27.9	4a
6´	1.29 (2H, *m*, CH_2_)	29.2	4a, 7´
7´	1.34 (2H, *m*, CH_2_)	27.9	6´
8´	1.43 (2H, *m*, CH_2_)	29.7	6´, 8a
8a	4.90 (1H, *m*, CH)	76.9	-

^a^ Proton was measured at 600 MHz

^b^ Carbon was measured at 150 MHz

White oil; Yield: 0.005%, [α] −16 (*c* 0.05, CHCl_3_). TLC: (*n*-hexane: EtOAc, 80:20 v/v): R_f_ = 0.56. UV (MeOH) λ_max_ (log €): 356 (3.80), 315 (4.03), 280 (4.32) nm. IR ν_max_ (CHCl_3_); 2921, 2853 (CH), 1759 (OC = O), 1463 cm^-1^. HRESIMS m/z [M+Na] 465.3240 (10), 338.3380 (90), 339.3430 (60) (calcd for C_30_H_50_O_2_, 442.7066). ^1^H NMR (CDCl_3_, 600MHz) δ ppm: 0.89 (3H, *m*, H-21), 1.25 (2H, *m*, H-18), 1.28 (14H, *m*, H-11, H-12, H-13, H-14, H-15, H-16, H-17), 1.29 (2H, *m*, H-6´), 1.30 (2H, *m*, H-19), 1.32 (2H, *m*, H-20), 1.34 (2H, *m*, H-7´), 1.43 (2H, *m*, H-8´), 1.51 (2H, *m*, H-2), 1.55 (2H, *m*, H-10), 1.59 (2H, *m*, H-5´), 2.02 (2H, *m*, H-9), 2.21 (2H, *m*, H-3), 2.25 (2H, *m*, H-1), 2.91 (2H, *m*, H-6), 2.93 (1H, *m*, H-4a), 4.90 (1H, *m*, H-8a), 5.33 (1H, *m*, H-7), 5.35 (1H, *m*, H-8), 5.40 (2H, *m*, H-4, H-5), 6.98 (1H, *m*, H-4´). ^13^C NMR (CDCl_3_, 150 MHz) δ ppm: 14.1 (C-21), 22.4 (C-20), 22.7 (C-18), 24.3 (C-3), 25.2 (C-1), 26.6 (C-2), 27.2 (C-7´), 27.4 (C-9), 27.9 (C-5´), 28.0 (C-10), 29.2 (C-6´), 29.5 (C-11, C-12, C-13, C-14), 29.6 (C-15, C-16, C-17), 29.7 (C-8´), 32.0 (C-19), 56.8 (C-4a), 57.3 (C-6), 76.9 (C-8a), 128.3 (C-8), 129.8 (C-7), 131.0 (C-4, C5), 134.4 (C-3´), 148.8 (C-4´), 173.9 (C-2´).

### Cytotoxic Effect of Pulchrin A

**Fig 2:**
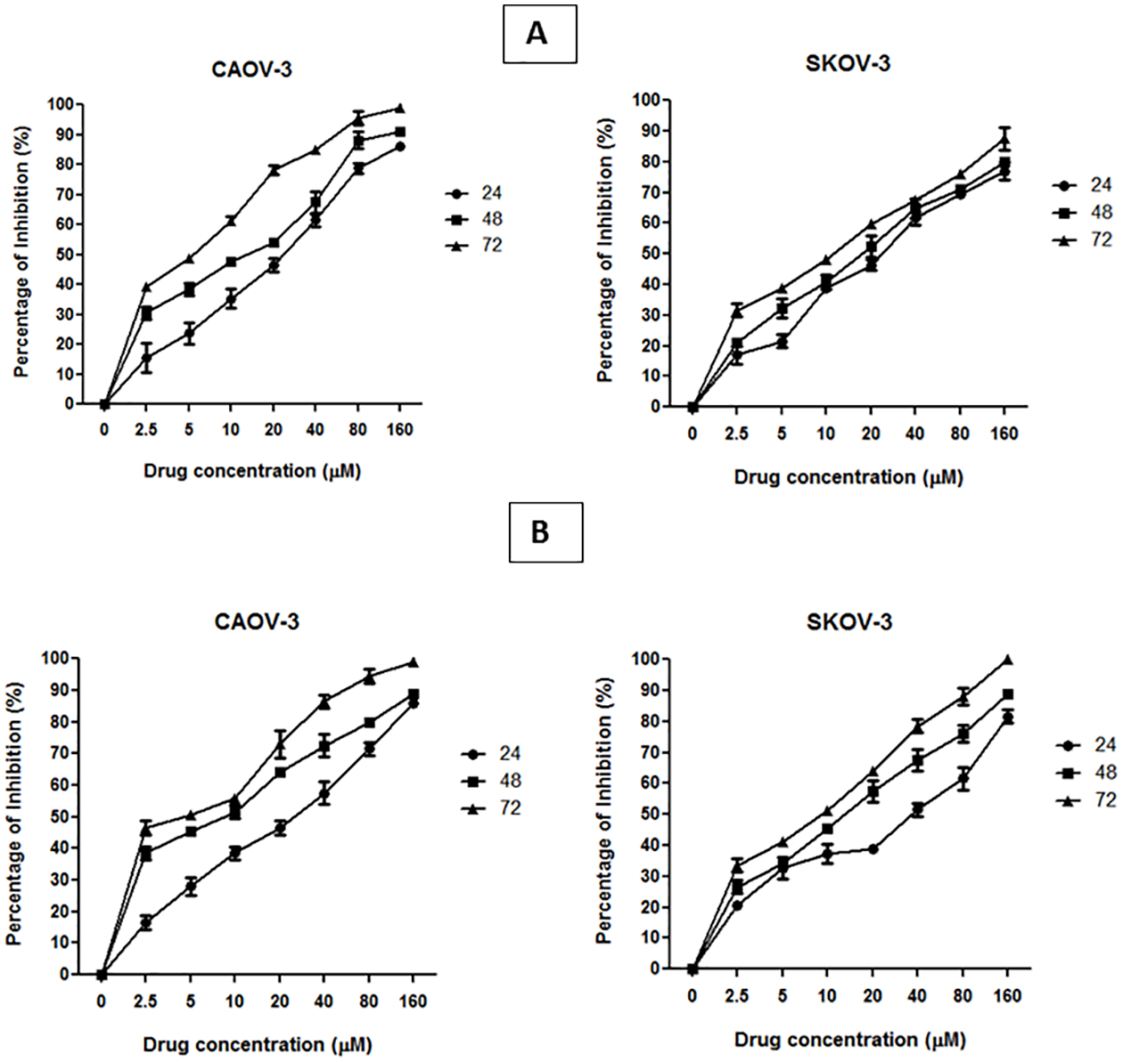
Inhibition curve of pulchrin A and cisplatin on ovarian cancer cell lines (CAOV-3 and SKOV-3) for 24, 48 and 72 h.

Three cell lines were tested, including two ovarian cancer cells (CAOV-3 and SKOV-3) and an immortalized human normal ovarian epithelial cell line (T1074) to determine the cytotoxic effects of pulchrin A (Table 2). In addition, cisplatin was also tested as positive control in this study (Fig 2). The IC_50_ results showed that pulchrin A inhibited 50% of CAOV-3 cells growth at 22.31 μM, as compared to 33.63 μM against SKOV-3 cells after 24 h of treatment. The CAOV-3 and SKOV-3 cells were further investigated for cytotoxic effects at 48 and 72 h, exhibiting decreases in the IC_50_ values as shown in Fig 2. Meanwhile, the cytotoxic effects of pulchrin A exhibited comparable effects compared with cisplatin at 24 h against CAOV-3 and SKOV-3 cells, which demonstrated that pulchrin A exerted higher cytotoxicity against both ovarian cancer cell lines. In contrast, a less cytotoxic effect against T1074 cells was found even at concentrations of more than 100 μM.

**Table 2:** Effect of pulchrin A and control drugs on cancer and normal cells at 24 h by MTT assay. The experiment was done in triplicate. Data are reported as mean ± SD.

Compounds	Cell lines	IC_50_ (μM)		
		24 h	48 h	72 h
**Pulchrin A**	CAOV-3	22.31 ± 0.42	19.25 ± 0.64	5.35 ± 0.21
	SKOV-3	31.63 ± 0.35	20.21 ± 0.21	13.03 ± 0.18
	T1074	>100	>100	>100
**Cisplatin**	CAOV-3	30.56 ± 0.33	10.17 ± 0.16	6.04 ± 0.09
	SKOV-3	42.50 ± 0.28	13.06 ± 0.11	9.20 ± 0.81
	T1074	>100	>100	>100

### Cytomorphology Evaluation of Apoptosis by AO/PI Double Staining

**Fig 3:**
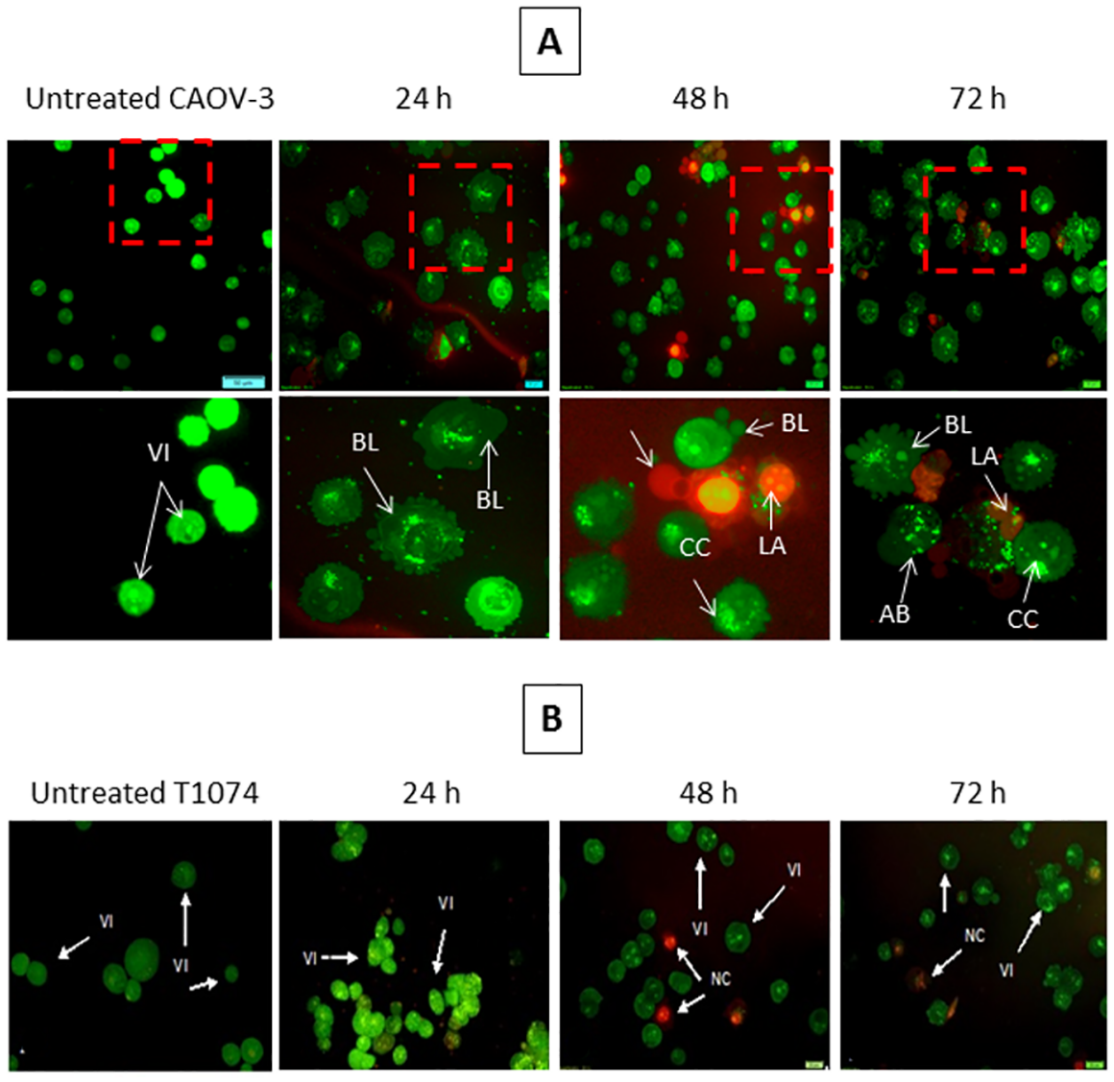
Micrographs of acridine orange (AO) and propidium iodide (PI) double-stained against untreated and treated CAOV-3 and T1074 cells with pulchrin A at IC_50_ concentration in a time-dependent manner. [A] Untreated and treated-CAOV-3 cells with pulchrin A at 24, 48 and 72 h of treatment. The CAOV-3 cells exhibited membrane cell blebbing as early as 24 h of treatment. The following 48 h of treatment showed more membrane cell blebbing and the presence of apoptotic bodies and cromatin condensation. The presence of orange staining cells at 72 h of treatment, representing the hallmark of late apoptosis. [B] Untreated and treated-T1074 cells with pulchrin A at 24, 48 and 72 h of treatment. The T1074 cells showed no significant differences in the cells morphology after treated with pulchrin A up to 72 h of treatment. VI, viable cells; BL, blebbing of the cell membrane; CC, chromatin condensation; LA, late apoptosis; AB, apoptotic bodies. (magnification 40× and 100×).

The apoptotic effect of pulchrin A on CAOV-3 and T1074 cells were observed via morphological changes under a fluorescence microscope. The cytomorphology evaluation was conducted in CAOV-3 cells due to pulchrin A showed low IC_50_ concentration against CAOV-3 cells compared with SKOV-3 cells in the cytotoxicity assay. After treatment at a concentration of 22 μM for 24, 48 and 72 h, the CAOV-3 cells exhibited apoptotic characteristics compared with T1074 cells. Under untreated conditions, the cells exhibited a healthy rounded shape with an intact nuclear structure. The cells morphology were changed after 24 h of treatment in CAOV-3 cells with the presence of cell membrane blebbing and DNA fragmentation. Extensive cell damage can be clearly observed after 48 h onwards with the presence of apoptotic bodies and a reddish-orange color, indicating that the cells underwent late apoptosis (Fig 3a). In contrast, the T1074 cells exhibited no significant difference on cell morphology compared with untreated T1074 cells towards apoptosis process, suggesting that pulchrin A was harmless to the normal ovarian cells (Fig 3b).

### Analysis of Membrane Alteration

**Fig 4:**
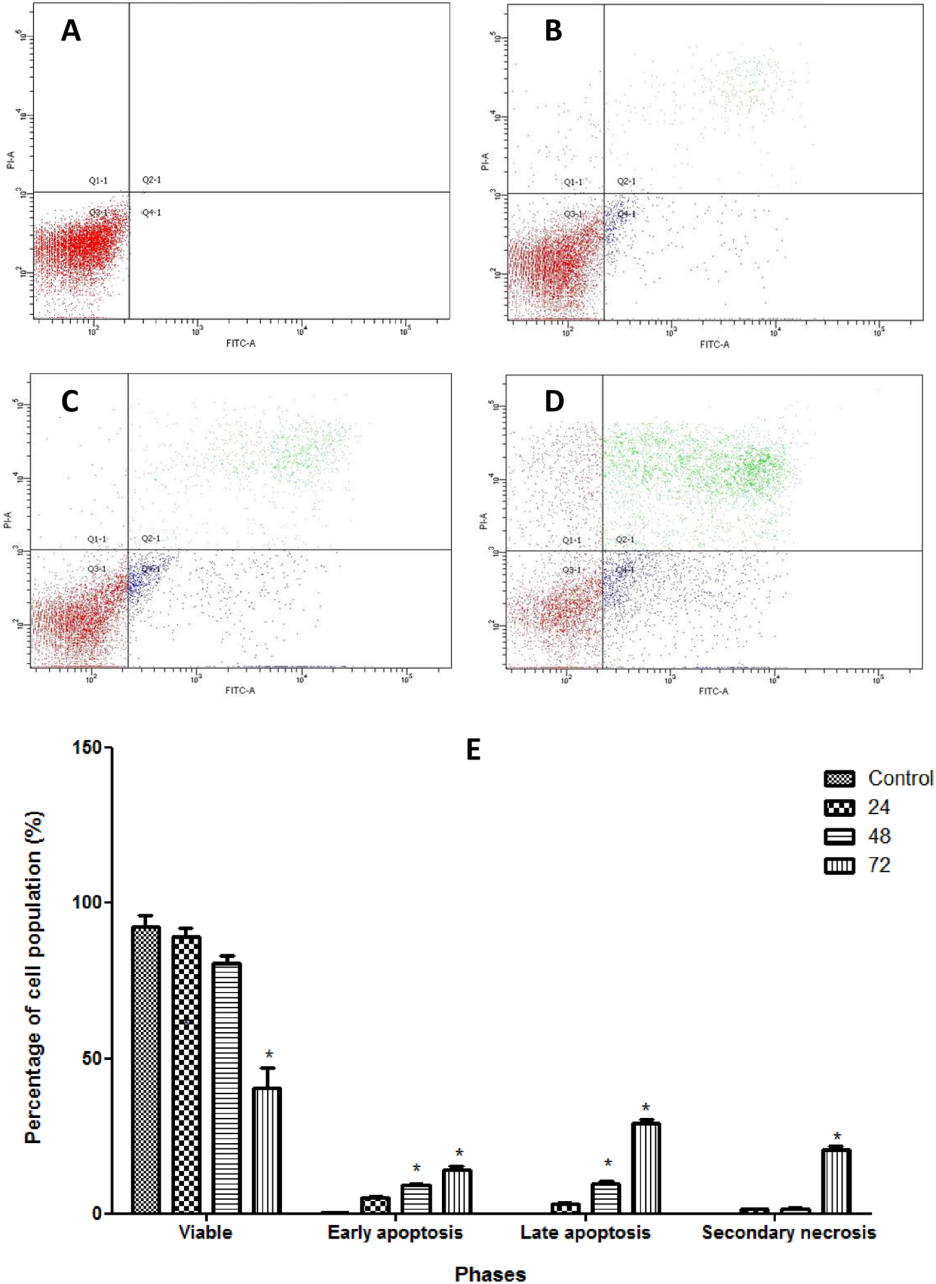
Analysis of Annexin V-FITC in CAOV-3 cells treated with pulchrin A in timely-dependent manner. [A] Control (untreated), [B] 24 h, [C] 48 h, [D] 72 h. Dark red, blue, green and light red color indicated viable, early apoptosis, late apoptosis and secondary necrosis, respectively. Movement of the cell population was started in Q3 (viable cells) to Q4 (early apoptosis) to Q2 (late apoptosis), and finally to Q1 (secondary necrosis). [E] Histogram of the cell populations of viable, early-apoptosis, late-apoptosis and secondary-necrosis CAOV-3 cells. Results were represented as mean ± SD of three replicates. *p<0.05 indicates significant difference from control.

Changes in cell membrane was observed by Annexin V-FITC assay conducted using a 25 mL flask cointaining CAOV-3 cells treated with pulchrin A at 24, 48 and 72 h. As shown in Fig 4, the results revealed that CAOV-3 cells treated with pulchrin A at 24, 48 and 72 h underwent early phase apoptosis as indicated by 5.3, 9.4 and 14.3% increases in quantity in a time-dependent manner, respectively. The cells that underwent late phase apoptosis and necrosis also increased within the first 24 to 72 h after treatment, unlike the viable cells, which showed a 92.5% to 40.5% decrease in cell population after treatment.

### Analysis of Caspases

**Fig 5:**
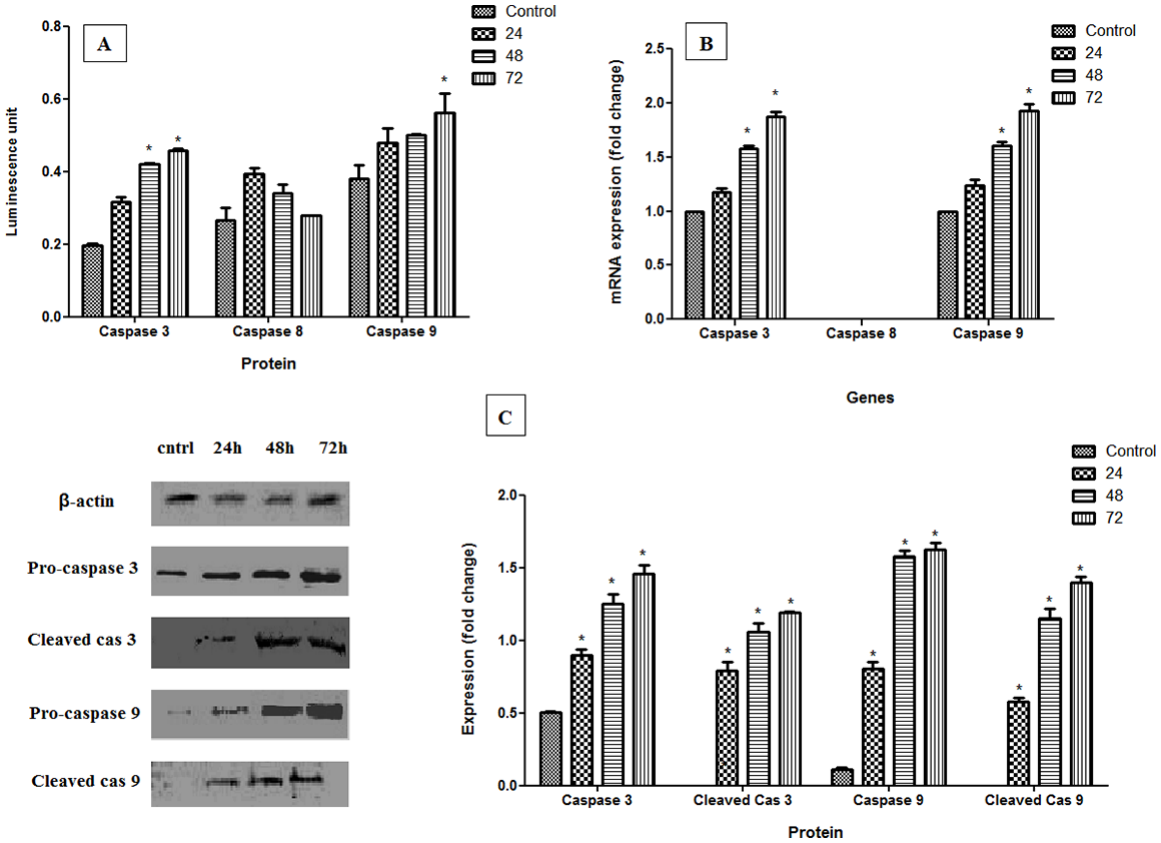
Involvement of caspases on CAOV-3 cells after treatment with pulchrin A for 24, 48 and 72 h of treatment. [A and B] Colorimetric and real-time PCR analysis of caspases 3, 8 and 9, respectively. [C] Western blot analysis characterized by images and histogram for pro-caspases and cleaved caspases. Results were represented as mean ± SD of three independent experiments. The significant difference is expressed as * *p*<0.05.

Caspases 3, 8 and 9 were tested to determine the apoptosis pathways. The results in Fig 5 show that caspases 9 and 3 were activated by pulchrin A exposure through the intrinsic and execution pathways, respectively. In contrast, caspase 8 showed irregular values, as depicted in the bar chart, suggesting that no activation of caspase 8 was detected via the stimulation of pulchrin A. Activation of caspases 3, 8 and 9 were also confirmed at the mRNA levels in which only caspases 3 and 9 exhibited over-expression relative to caspase 8 in a time-dependent manner. Similarly, the expression levels of caspase 3 and caspase 9 proteins were significantly upregulated (*p*<0.05) relative to that of the untreated CAOV-3 cells. In addition, the protein expression of cleaved caspase 3 and cleaved caspase 9 increased under the three different treatment periods, indicating that induction of apoptosis occurred via the intrinsic and execution pathways.

### Analysis of Mitochondrial Changes

**Fig 6:**
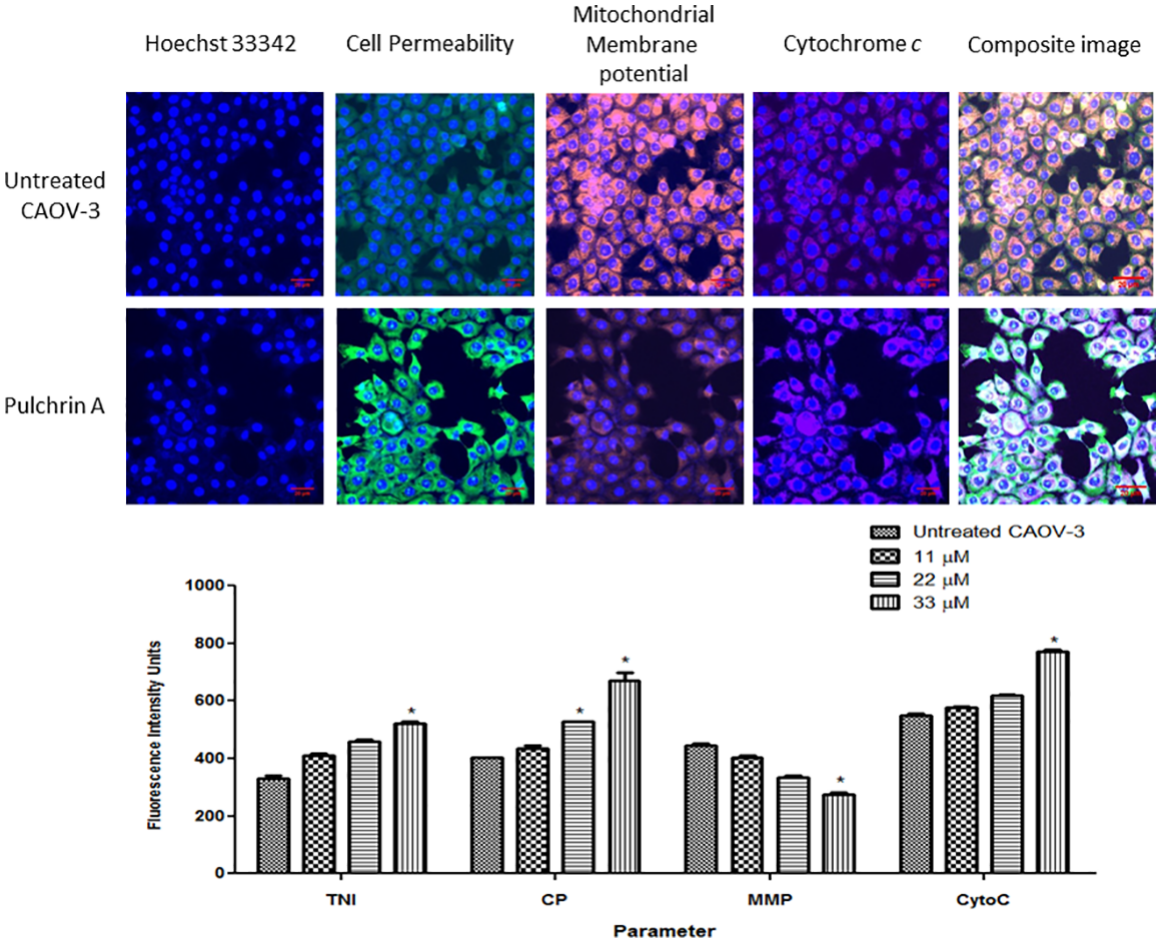
Fluorescence imaging and quantitative analysis of CAOV-3 cells treated with pulchrin A and untreated CAOV-3 cells against mitochondrial parameters. The cells were stained with Hoechst, cell permeability, mitochondrial membrane potential (MMP) and cytochrome *c* dyes. The images on each row were captured from the same field. The CAOV-3 cells exhibited a reduction in cell number and MMP, whereas the cells treated with pulvhrin A at 24 h (20× magnification) showed increases in cell permeability and cytochrome *c* release. Histogram represents average intensities observed simultaneously in CAOV-3 cells in a concentration-dependent manner for total nuclear intensity, cell permeability, MMP and cytochrome *c* release. All data were determined as mean ± SD in which the significant difference was expressed as *p< 0.05.

This study was performed to examine the involvement of mitochondria in apoptosis upon pulchrin A treatment. The three parameters of total nuclear intensity, cell permeability and cytochrome *c* release indicated that fluorescence intensity was increased, as shown in Fig 6. The fluorescence intensities of these three parameters increased at concentrations as low as 22 μM and significant differences (*p*<0.05) were determined at a concentration of 33 μM. In contrast, the fluorescence intensity of the mitochondrial membrane potential (MMP) for the pulchrin A-treated CAOV-3 cells decreased in a concentration-dependent manner. Significant difference was observed between the treated and untreated CAOV-3 cells at concentrations higher than 33 μM when *p*<0.05.

### DNA Fragmentation Assay

**Fig 7:**
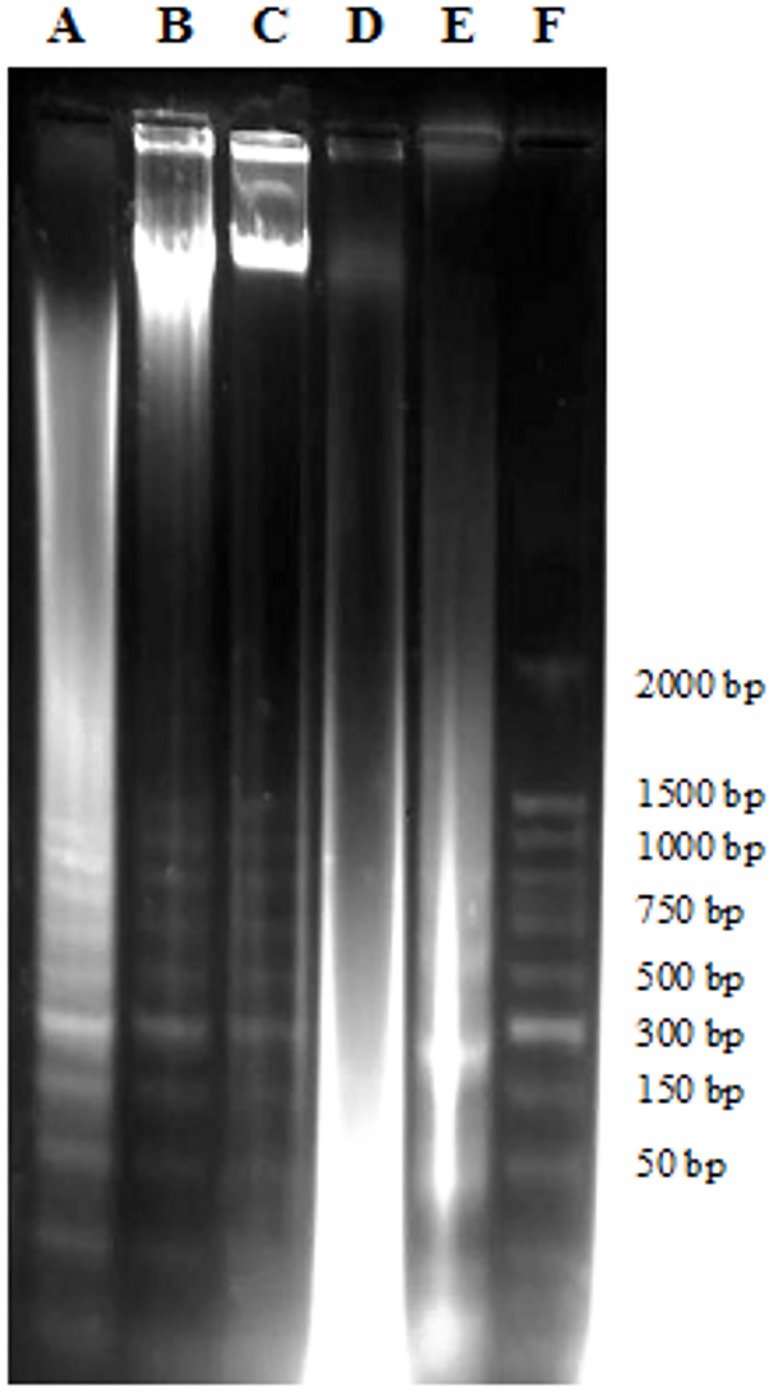
DNA fragmentation of untreated and treated CAOV-3 cells with pulchrin A (22 μM) by electrophoretic separation. Lanes A-C: cells treated with pulchrin A for 72, 48 and 24 h, respectively. Lane D: untreated cells. Lane E: positive control of treated-HL-60 cells with actinomycin D. Lane F: DNA marker (50 base pair).

This assay was conducted to confirm the occurrence of late apoptosis in cells treated with pulchrin A. The presence of a DNA ladder on the CAOV-3 cells was observed after 24 h of treatment (Fig 7); the same was observed for the next 48 and 72 h. The formation of DNA ladder proved the occurrence of DNA fragmentation in the CAOV-3 cells, which induced apoptosis.

### Evaluation of Bax, Bcl-2 and Survivin

**Fig 8:**
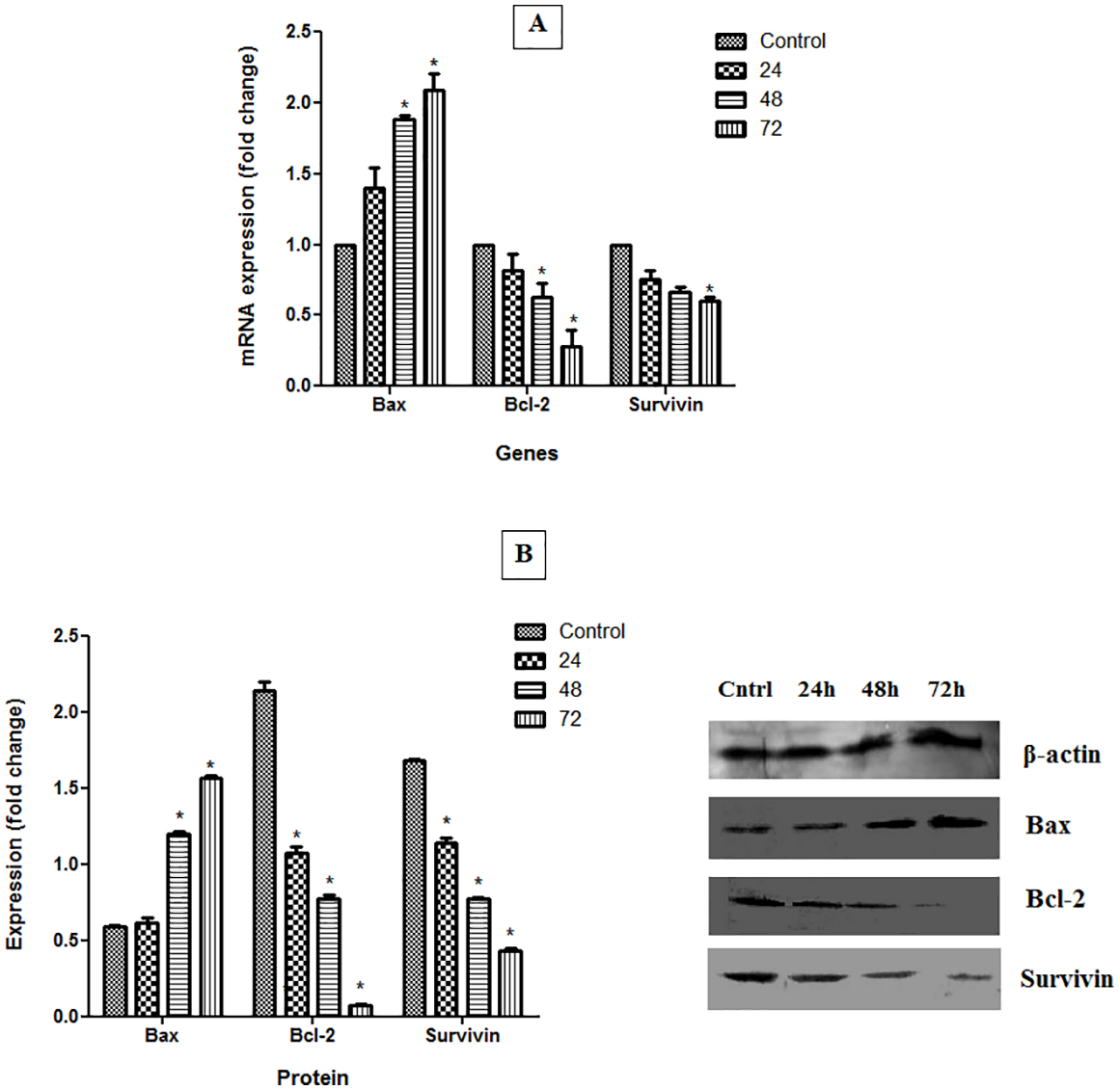
Ability of pulchrin A (22 μM) to induce apoptosis on the gene and protein levels for Bax, Bcl-2 and survivin with *β*-actin used as a loading control. CAOV-3 cells were treated with pulchrin A at 24, 48 and 72 h. All data were expressed as mean ± standard deviation (SD). Statistical significance was expressed at *p< 0.05.

The expression levels of Bax, Bcl-2, as well as survivin mRNAs and proteins in treated and untreated CAOV-3 cells are presented in Fig 8. Bax mRNA and protein were over expressed in cells treated with pulchrin A. In contrast, the expression levels of Bcl-2 and survivin in both mRNAs and proteins were down-regulated under all treatment periods relative to that in the untreated CAOV-3 cells. Significant differences (*p*<0.05) in the expression levels of mRNAs and proteins expression were observed in cells treated with pulchrin A for 48 and 72 h for Bax and Bcl-2, and 72 h for survivin.

### Apoptotic Protein Markers Analyses

**Fig 9:**
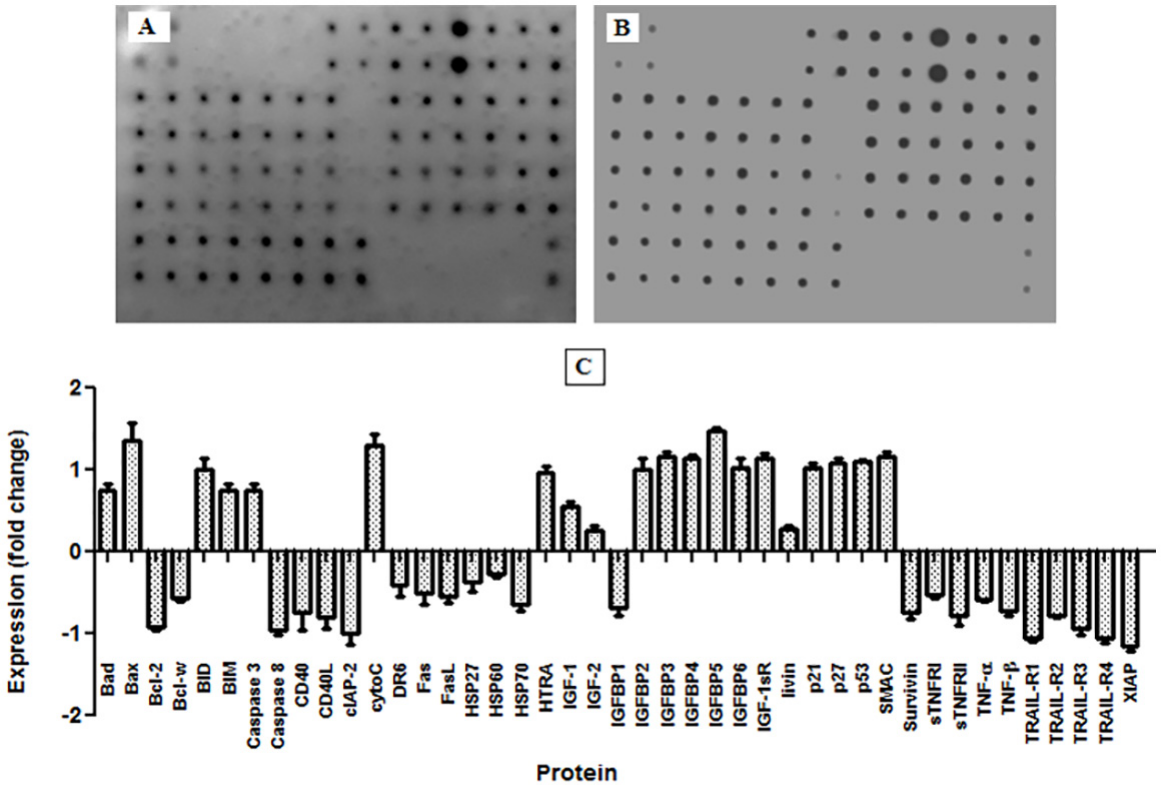
Human apoptosis proteome profile array in CAOV-3 cells treated with pulchrin A for 24 h. [A] Expression of apoptotic proteins in untreated cells. [B] Expression of apoptotic proteins upon pulchrin A treatment. [C] Histogram of quantitative analysis between untreated and treated cells, with the positive and negative fold changes indicating up-regulation and down-regulation of protein expression, respectively. Results expressed as mean ± SD at *p<0.05 were considered significant.

By analysis of the protein profile array, 43 proteins were found to be involved in apoptosis, as shown in Fig 9. Of these proteins, 20 consisted of Bad, Bax, BID, BIM, caspase 3, cytoC, HTRA, IGF-1, IGF-2, IGFBP-2, 3, 4, 5 and 6, IGF-1 sR, livin, p21, p27, *p53* and SMAC were over expressed after treatment of CAOV-3 cells with pulchrin A. Meanwhile, the expression levels of the remaining 23 proteins were downregulated when treated with pulchrin A. The proteins found to be involved were bcl-2, bcl-w, caspase 8, CD40, CD40L, cIAP-2, DR6, Fas, FasL, HSP27, HSP60, HSP70, IGFBP-1, survivin, sTNFRI, sTNFRII, TNF-α, TNF-β, TRAIL-R1, R2, R3, R4 and XIAP.

### Cell Cycle Analysis

**Fig 10:**
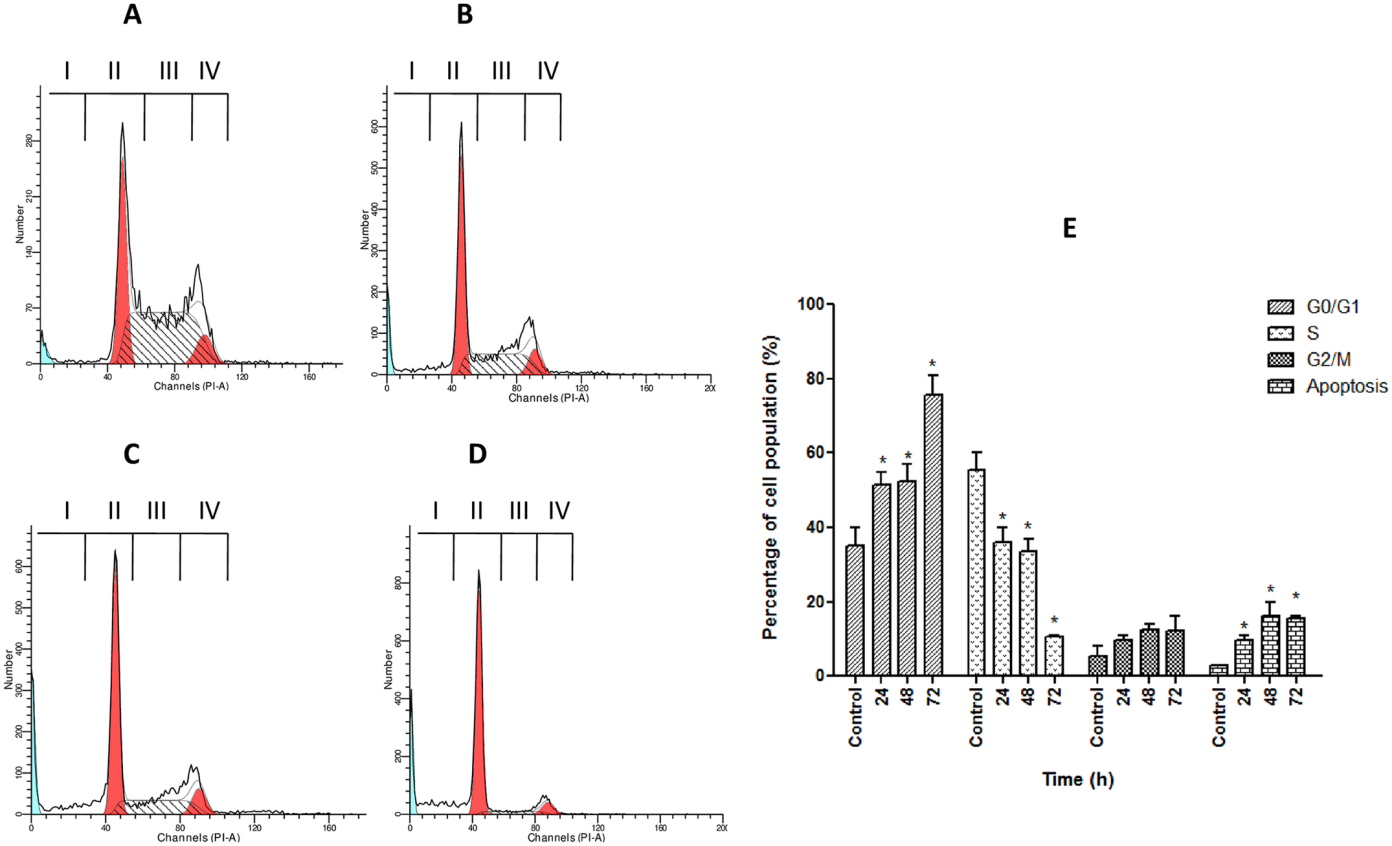
Analysis of cell cycle arrest in CAOV-3 cells treated with pulchrin A at three different time points. [A] untreated CAOV-3 cells and CAOV-3 cells treated with pulchrin A for [B] 24 h, [C] 48 h and [D] 72 h, as well as [E] graphical analysis of cell cycle arrest in CAOV-3 cells at the G0/G1 phase depicting percentage increases in the population of cells treated with pulchrin A at 24, 48 and 72 h. I, apoptotic cells; II, G0/G1 phase; III, S phase; IV, G2/M phase. G0/G1 is Gap 0 or 1 indicating the resting phase in which the cell has left the cycle and stopped dividing. G1 indicates that cells increase in size. S phase represents synthesis in DNA replication occurs, whereas G2/M denotes Gap2 or mitosis, in which the cell continues to develop. Results were expressed as mean ± SD of three replicates. (*) indicates significant difference from the control when *p*<0.05.

As shown in Fig 10, CAOV-3 cells underwent cell cycle arrest upon treatment with pulchrin A. A significant increase (*p*<0.05) relative to untreated cells was detected in the G0/G1 cell populations. The percentage of cell population increased by 34.3, 35.0 and 50.0% after 24, 48 and 72 h of pulchrin A exposure, respectively, corresponding to G0/G1 arrest. In contrast, the S phase of CAOV-3 cell cycle showed significantly decrease in cell percentage compared with untreated cells upon pulchrin A treatment in time-dependent manner of 37.0, 24.0, 22.3 and 3.0%, respectively. Meanwhile, the percentages of apoptotic cells in the sub G0/G1 region were also increased from 1.8% (untreated) to 10.3% (72 h).

### Docking of pulchrin A into ABT-737 binding site of Bcl-2

**Fig 11:**
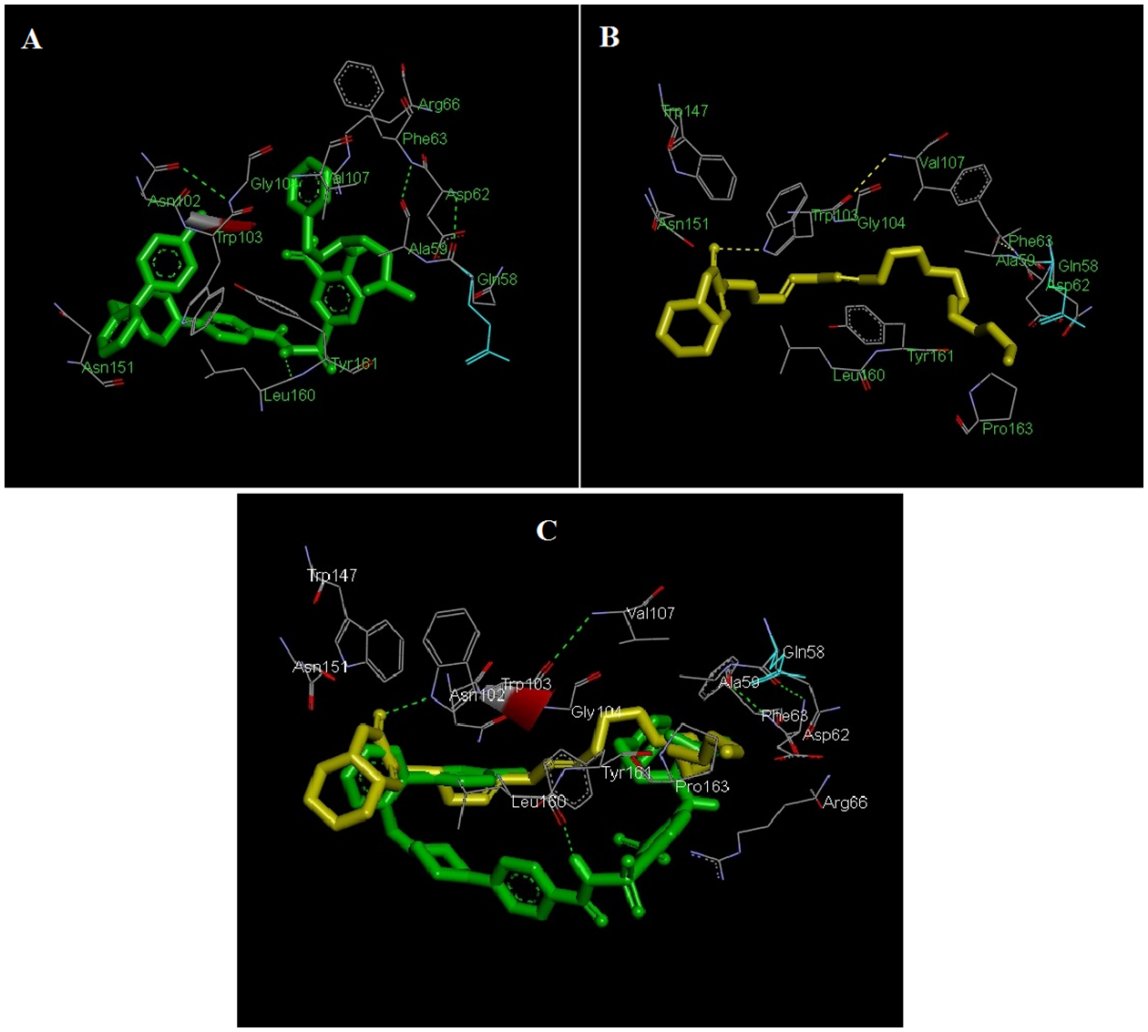
Docked complexes of pulchrin A at the ABT-737 binding site of Bcl-2. [A] Interaction between ABT-737 and Bcl-2. [B] Interaction between pulchrin A and Bcl-2. [C] 3-dimensional structure of pulchrin A (yellow) overlaid with the crystal structure of ABT-737 (green) complexed in Bcl-2. Hydrogen bonding is depicted by green dashed lines.

This experiment reflected the binding interaction between pulchrin A and Bcl-2 protein with the ABT-737 binding site. The selection of protein according to the activation of apoptotic pathways through caspases analyses is shown in Fig 11. The result showed that one intermolecular hydrogen bond was present in the formation of pulchrin A with Bcl-2 interactions at TRP-103 residue with -6.28 kcal/mol binding energy. Meanwhile, one intermolecular hydrogen bond that was detected at the LEU-160 of Bcl-2 with the ABT 737, with -7.92 kcal/mol binding energy.

## Discussion

Pulchrin A is a new natural compound that was isolated for the first time from natural products. The basic structure is classified in coumarin groups with many uses in modern medicine to treat various diseases such as Alzheimer’s disease as well as kidney and liver diseases [34, 35]. In particular, pulchrin A was identified using spectroscopic techniques with a complete analysis of NMR, IR and MS. The NMR of pulchrin A was recorded in chloroform-*d* (CDCl_3_-*d*) on a Bruker 400 MHz spectrometer. HRESIMS analysis showed that pulchrin A exhibited a [M+Na]^+^ peak at *m/z* 465.3240, which corresponds to the molecular formula of C_30_H_50_O_2_Na. The unsaturated lactone coumarin moiety was detected at the IR absorption bands of 1759 cm^-1^. The presence of this group was indicated by the ^1^H and ^13^C NMR resonance at δ 6.98 (H-4'), 2.93 (H-4a), and 4.90 (H-8a), as well as δ 173.9 (C-2'), 134.4 (C-3'), 148.8 (C-4'), 56.8 (C-4a) and 76.9 (C-8a) ppm. Meanwhile, the ^1^H NMR data showed the existence of methine protons at the hydrocarbon chain between δ 5.33 and 5.40 ppm. Furthermore, the distortionless enhancement by polarization transfer (DEPT) data confirmed the existence of twenty methylene (CH_2_) groups, seven methine (CH) groups and one methyl (CH_3_) groups corresponding to the whole skeletal framework. The protons and carbons were assigned and established using the heteronuclear single quantum coherence (HSQC) and heteronuclear multiple bond correlation (HMBC) spectroscopic spectra (Table 1). Thus, a new isolated compound was deduced as a pulchrin A. An in-depth evaluation of this compound was conducted to determine new anticancer agents that would help overcome the recent problem related to resistance to ovarian cancer drugs [36]. Pulchrin A was investigated for its capability to initiate apoptosis in CAOV-3 and SKOV-3 cells. According to Table 2, pulchrin A exhibited stronger cytotoxicity effects on CAOV-3 cells at concentration as low as 22 μM upon 24 h of exposure. The compound also exerted better effects than cisplatin (the control drug). This result demonstrates that pulchrin A can be used as a principal alternative drug for treating ovarian cancer either individually or synergistically.

The ability of pulchrin A to induce apoptosis was proven by several apoptotic markers studied widely, including morphological changes in cells and nuclei, exposure of phosphatidylserine (PS) on the cell surface, as well as DNA fragmentation [37]. Changes in CAOV-3 cells morphology occurred because of the reaction of the apoptotic signal stimulated by pulchrin A. In fact, the term “apoptosis” refers to changes in cell morphology [38, 39], which are associated with several morphological characteristics such as plasma membrane blebbing caused by activation of gelsolin [40], karyorrhexis, in which the nuclear content is irregularly distributed within the cytoplasm, cleavage of chromatin, development of apoptotic bodies due to cleavage of PAK2 [41] and opening of cell surface for phagocytosis [42, 43]. All these alterations in cells undergoing apoptosis were observed in the CAOV-3 cells treated with pulchrin A for 24, 48 and 72 h (Fig 3a).

Apoptotic cells are exposed to phagocytosis because of loss of integrity of the plasma membrane surface. This condition is caused by PS, which is previously located in the leaflet of the plasma membrane moving toward the outer leaflet because of inhibition of translocases (flippases) and activation of scramblases [44]. PS exposure allows the engulfment or 'eat me' signal, which is recognized by phagocytes [45, 46]. Therefore, detection of PS in response to apoptosis is important because it is the universal marker for early apoptosis [47]. As shown in Fig 4, Annexin V, which is a soluble molecule detected the exposed PS in the CAOV-3 cells and was recorded by flowcytometry on the first day of pulchrin A treatment.

The involvement of caspases in this study was the strongest determinant of apoptosis-dependent pathways because of the proteolytic activity of caspases can mediate apoptotic cell death [48–50]. Through the proteolytic cascade, activation of caspase can induce other caspases, leading to activation of the apoptotic signaling pathway [51]. Two caspases were activated by pulchrin A in this study, caspases 3 and 9, in the execution and intrinsic pathways, respectively. Furthermore, proteolytic activity in caspases were able to cleave proteins at aspartic acid residues, as shown in Fig 5. The involvement of caspase 9 as the initiator caspase in the intrinsic or mitochondrial pathway was also clarified by the morphological changes that occurred in CAOV-3 cells, as discussed above. Once caspases are initiated, a permanent involvement towards cell death is observed. A recent study by Nordin et al. demonstrated that liriodenine isolated from *E*. *pulchrum* also activated the intrinsic pathway and the execution pathways [52].

Activation of intrinsic pathway was also characterized by the disruption of the mitochondrial role. The intrinsic pathway is closely associated with the permeabilization of the external mitochondrial membrane through the Bcl-2 family [53, 54] which is regulated by the *p53* protein [55]. A current study found that pulchrin A stimulated the expression of pro-apoptotic proteins such as Bax, Bad, BIM and BID. Meanwhile, pulchrin A suppressed the anti-apoptotic proteins, including the Bcl-2 and Bcl-w proteins in the CAOV-3 cells, leading to apoptosis. This effect causes a deficiency in mitochondrial membrane potential (MMP), resulting in cytochrome *c* release into the cytoplasm [56] as shown in Fig 6. In addition, cytochrome *c*, second mitochondrial-derived activator of caspase (SMAC), high temperature requirement A (HTRA), apoptosis inducing factor (AIF) and endonuclease G were released into the cytosol upon mitochondrial distraction [57]. Therefore, SMAC and HTRA were also over expressed in the study of proteome profile array.

When apoptogenic factors are released into the cytosol, which are initially in mitochondria, the downstream destruction programs are activated [58] by promoting caspase 3 activation or by acting as caspase-independent death effectors [59–61]. The phenomenon was demonstrated by the increase in caspase 3 levels in the CAOV-3 cells after treatment with pulchrin A. Activation of executioner phase by caspase 3 is identified by the formation of nuclear fragmentation [62]. This fragmentation was indicated by the formation of a DNA ladder in CAOV-3 cells after treatment with pulchrin A for 24, 48 and 72 h (Fig 7). According to Wylie [63], DNA ladder is often used as a marker to determine apoptosis, which is created through DNA cleavage in nucleosomes to form fragments with an average length of 180 base pairs. Thus, activation of caspase 3 is important to mediate the response that occurs in the nuclei of cells.

Expression of survivin has commonly been demonstrated in apoptosis-related ovarian cancer research. In the event of cancer, survivin is stimulated at high rates, inhibiting the apoptosis of cancerous cell [64–66]. Studies on pulchrin A treatment using CAOV-3 cells showed that down-regulation of survivin demonstrates cell apoptosis. In addition, survivin which is also involved in the regulation of caspases 3, 7 and 9 in cancer cells was inhibited upon drug treatment [67, 68]. This result shows similarity with the effect of pulchrin A by inhibiting the action of survivin in the CAOV-3 cells.

Pulchrin A has also been shown to inhibit the CAOV-3 cell cycle from further division. In general, cancer cells can divide continuously if no interruption in the cell cycle occurs [69]. However, pulchrin A successfully interrupted the CAOV-3 cell cycle at the G0/G1 phase (Fig 10). This condition is caused by the activation of the cyclin-dependent kinase (CDK) inhibitor protein that inhibits the cell cycle [70], thereby inhibiting the proliferation of cells. In the present study, the proliferation of CAOV-3 cells was arrested because of over-expression of p21, p27 and p53 proteins, compared with the untreated cells (Fig 9). The p21 and p27 inhibitors inactivated the G1 CDK-cyclin complexes, thereby arresting the CAOV-3 cell cycle at the G0/G1 phase. Meanwhile, expression of p21 is under transcriptional control of the p53 protein [71], in which the expression of p53 correlates with the production of p21. Thus, up-regulation of the expression levels of p21, p27 and p53 can prove the existence of barriers on CAOV-3 cell proliferation, ultimately leading to apoptosis [72].

The ability of pulchrin A to trigger apoptosis was also observed through the binding interaction between pulchrin A and the Bcl-2 protein, which was directly involved in the activation of the mitochrondrial pathway. From the findings, we propose that pulchrin A is a potential Bcl-2 antagonist because of its comparable binding energies exhibited with ABT-737. This situation could explain the induction of apoptosis in CAOV-3 cells. Previous studies have also shown that the interaction between N-heteroaryl sulfonamides in the BH3 domain of the Bcl-2 protein overlaid with ABT 737 prompted the discovery of potent inhibitors [30]. The analysis of pulchrin A bound with Bcl-2 at the ABT-737 binding site suggests that pulchrin A shares a similar binding motif with ABT-737 in the BH3 domain of Bcl-2 and thus promotes apoptosis in human ovarian cancer cells.

## Conclusion

To our knowledge, pulchrin A was isolated as a new natural compound exhibiting apoptogenic effects against ovarian cancer cells. The discovery of pulchrin A as a promising lead can be used to develop potent and specific agents for cell cancer inhibitors and to overcome resistivity against chemotherapeutics. However, continued efforts to prove the ability of pulchrin A targeting Bcl-2-regulated apoptosis as well as further *in vivo* animal studies should be conducted to provide more evidence to the anticancer effect of pulchrin A on ovarian cancer.

## Supporting Information

S1 Fig The ^1^H-NMR spectrum of pulchrin A. (TIF)

S2 Fig The ^13^C-NMR spectrum of pulchrin A. (TIF)

S3 Fig The DEPT spectrum of pulchrin A. (TIF)

S4 Fig The COSY spectrum of pulchrin A. (TIF)

S5 Fig The HSQC spectrum of pulchrin A. (TIF)

S6 Fig The HMBC spectrum of pulchrin A. (TIF)

S7 Fig The HPLC chromatogram [A] and mass spectrum [B] of pulchrin A. (TIF)
