# Expression of Concern: Pulchrin A, a New Natural Coumarin Derivative of *Enicosanthellum pulchrum*, Induces Apoptosis in Ovarian Cancer Cells via Intrinsic Pathway

**DOI:** 10.1371/journal.pone.0342604

**Published:** 2026-02-12

**Authors:** The PLOS One Editors

After this article [1] was published, concerns were raised regarding results presented in Fig 8. Specifically, the Fig 8B Survivin panel in this article [1] appears similar to the Fig 8 Bcl-2 panel in [2].

First author NN stated that an error occurred during figure preparation but did not specify which, if either panel, is correct. They also stated that the original data underlying the results in [1] are no longer available. Contrary to the declaration in the Data Availability statement, the original raw data files supporting the article’s results were not provided with the article, and the authors have not provided these data following editorial request. As the original raw data files supporting the article’s results have not been made available, this article [1] does not comply with the PLOS Data Availability policy.

The *PLOS One* Editors issue this Expression of Concern to inform readers of the above unresolved concerns.

The Fig 8B Survivin panel reports material similar to that in [2], published in 2015 by Dovepress, under a CC-BY-NC 3.0 license. The Fig 8B Survivin panel is not offered under a CC BY license and is therefore excluded from this article’s [1] license.

## References

[1] Nordin N, Fadaeinasab M, Mohan S, Mohd Hashim N, Othman R, Karimian H, et al. Pulchrin A, a new natural coumarin derivative of *Enicosanthellum pulchrum*, induces apoptosis in ovarian cancer cells via intrinsic pathway. PLoS One. 2016;11(5):e0154023. doi: 10.1371/journal.pone.0154023 27136097 PMC4852948

[2] Nordin N, Majid NA, Hashim NM, Rahman MA, Hassan Z, Ali HM. Liriodenine, an aporphine alkaloid from *Enicosanthellum pulchrum*, inhibits proliferation of human ovarian cancer cells through induction of apoptosis via the mitochondrial signaling pathway and blocking cell cycle progression. Drug Des Devel Ther. 2015;9:1437–48. doi: 10.2147/DDDT.S77727 25792804 PMC4362660

