# Supplementary figures and images for: Pulchrin A, a New Natural Coumarin Derivative of Enicosanthellum pulchrum, Induces Apoptosis in Ovarian Cancer Cells via Intrinsic Pathway
Source: PLoS One. 2016 May 2;11(5):e0154023. doi: 10.1371/journal.pone.0154023 (PMC4852948; doi:10.1371/journal.pone.0154023)

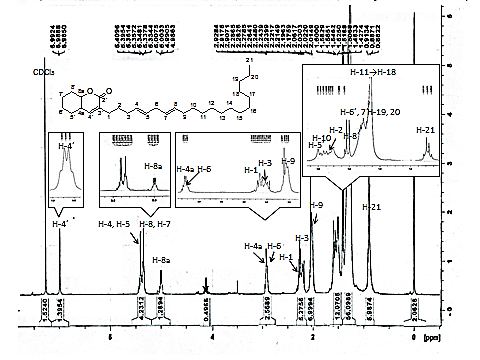

Supplement: S1 Fig — (TIF) [file pone.0154023.s001.tif]

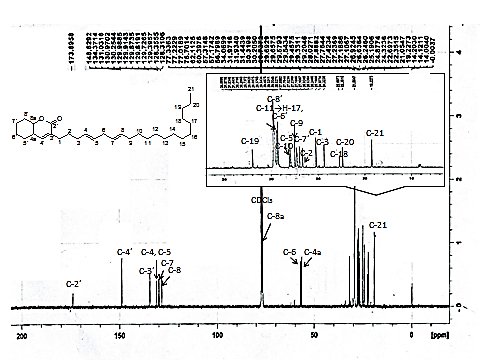

Supplement: S2 Fig — (TIF) [file pone.0154023.s002.tif]

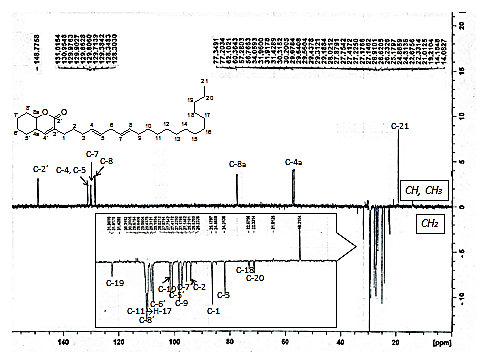

Supplement: S3 Fig — (TIF) [file pone.0154023.s003.tif]

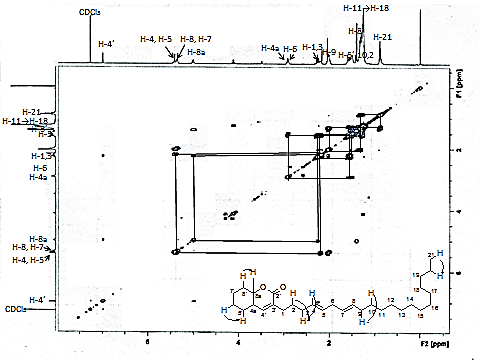

Supplement: S4 Fig — (TIF) [file pone.0154023.s004.tif]

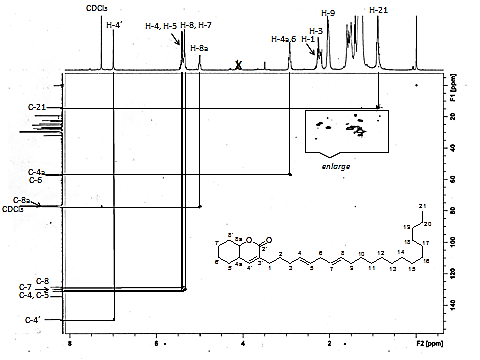

Supplement: S5 Fig — (TIF) [file pone.0154023.s005.tif]

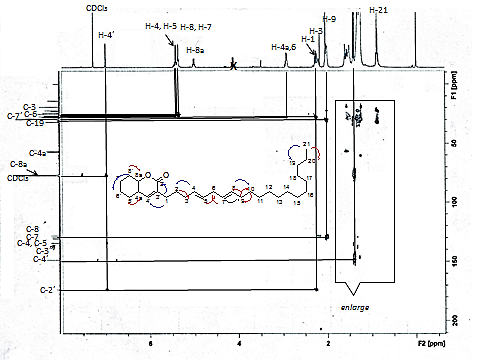

Supplement: S6 Fig — (TIF) [file pone.0154023.s006.tif]

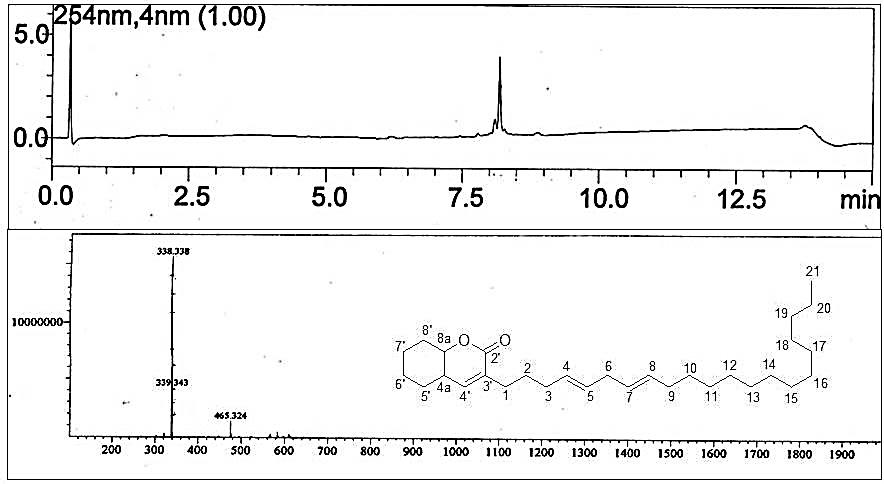

Supplement: S7 Fig — (TIF) [file pone.0154023.s007.tif]
